# SiCILIA—Silicon Carbide Detectors for Intense Luminosity Investigations and Applications

**DOI:** 10.3390/s18072289

**Published:** 2018-07-15

**Authors:** Salvatore Tudisco, Francesco La Via, Clementina Agodi, Carmen Altana, Giacomo Borghi, Maurizio Boscardin, Giancarlo Bussolino, Lucia Calcagno, Massimo Camarda, Francesco Cappuzzello, Diana Carbone, Salvatore Cascino, Giovanni Casini, Manuela Cavallaro, Caterina Ciampi, Giuseppe Cirrone, Giacomo Cuttone, Alberto Fazzi, Dario Giove, Giuseppe Gorini, Luca Labate, Gaetano Lanzalone, Grazia Litrico, Giuseppe Longo, Domenico Lo Presti, Marco Mauceri, Roberto Modica, Maurizio Moschetti, Annamaria Muoio, Franco Musumeci, Gabriele Pasquali, Giada Petringa, Nicolò Piluso, Giacomo Poggi, Stefania Privitera, Sebastiana Puglia, Valeria Puglisi, Marica Rebai, Sabina Ronchin, Antonello Santangelo, Andrea Stefanini, Antonio Trifirò, Massimo Zimbone

**Affiliations:** 1 Istituto Nazionale di Fisica Nucleare (INFN), Laboratori Nazionali del Sud (LNS), Via S. Sofia 62, 95123 Catania, Italy; francesco.lavia@imm.cnr.it (F.L.V.); agodi@lns.infn.it (C.A.); altana@lns.infn.it (C.A.); cappuzzello@lns.infn.it (F.C.); carboned@lns.infn.it (D.C.); manuela.cavallaro@lns.infn.it (M.C.); cirrone@lns.infn.it (G.C.); cuttone@lns.infn.it (G.C.); lanzalone@lns.infn.it (G.L.); annamaria.muoio@lns.infn.it (A.M.); fmusumec@dmfci.unict.it (F.M.); giada.petringa@lns.infn.it (G.P.); puglia@lns.infn.it (S.P.); 2 Institute for Microelectronics and Microsystems (IMM), National Research Council (CNR), VIII Strada, 5, 95121 Catania, Italy; stefania.privitera@imm.cnr.it (S.P.); massimo.zimbone@imm.cnr.it (M.Z.); 3 Trento Institute for Fundamental Physics and Applications (TIFPA), National Institute for Nuclear Physics (INFN), Fondazione Bruno Kessler (FBK-Trento), Via Sommarive 14, 38123 Povo Trento, Italy; gborghi@fbk.eu (G.B.); boscardi@fbk.eu (M.B.); ronchin@fbk.eu (S.R.); 4 Istituto Nazionale di Ottica (INO), Consiglio Nazionale delle Ricerche (CNR), Via G. Moruzzi 1, 56124 Pisa, Italy; giancarlo.bussolino@ino.it (G.B.); luca.labate@ino.it (L.L.); 5 Istituto Nazionale di Fisica Nucleare (INFN)—Sezione di Catania, Italy; lucia.calcagno@ct.infn.it (L.C.); domenico.lopresti@ct.infn.it (D.L.P.); 6 Department of Physics and Astronomy, University of Catania, Via S. Sofia 64 Catania, Italy; 7 Paul Scherrer Institute, ODRA/116, 5232 Villigen, Switzerland; massimo.camarda@psi.ch; 8 STMicroelectronics, Stradale Primosole, 50, 95121 Catania, Italy; salvatore.cascino@st.com (S.C.); peppe.longo@st.com (G.L.); roberto.modica@st.com (R.M.); maurizio.moschetti@st.com (M.M.); nicolo.piluso@st.com (N.P.); valeria-sst.puglisi@st.com (V.P.); antonello.santangelo@st.com (A.S.); 9 Istituto Nazionale di Fisica Nucleare (INFN)—Sezione di Firenze, Via G. Sansone 1, 50019 Sesto Fiorentino, Italy; casini@fi.infn.it (G.C.); caterina.ciampi@stud.unifi.it (C.C.); pasquali@fi.infn.it (G.P.); poggi@fi.infn.it (G.P.); stefanini@fi.infn.it (A.S.); 10 Dipartimento di Fisica, Università di Firenze, Via G. Sansone 1, 50019 Sesto Fiorentino, Italy; 11 Istituto Nazionale di Fisica Nucleare (INFN)—Sezione di Milano, Department of Energy, Politecnico di Milano, Via Celoria 16, 20133 Milano, Italy; alberto.fazzi@polimi.it (A.F.); dario.giove@mi.infn.it (D.G.); 12 Istituto Nazionale di Fisica Nucleare (INFN)—Sezione di Milano Bicocca, Department of Physics, Università degli Studi di Milano-Bicocca, Piazza della Scienza 3, 20126 Milano, Italy; giuseppe.gorini@unimib.it (G.G.); rebai@ifp.cnr.it (M.R.); 13 Facoltà di Ingegneria e Architettura, Università Kore, Cittadella Universitaria, 94100 Enna, Italy; 14 LPE, XVI Strada, 95121 Catania, Italy; grazia.litrico@gmail.com (G.L.); Marco.Mauceri@lpe-epi.com (M.M.); 15 Dipartimento di Scienze MIFT dell’Universitá di Messina, V.le F. S. D’Alcontres 31, 98166 Massina, Italy; atrifiro@unime.it

**Keywords:** silicon carbide, nuclear and particle detector, radiation hardness

## Abstract

Silicon carbide (SiC) is a compound semiconductor, which is considered as a possible alternative to silicon for particles and photons detection. Its characteristics make it very promising for the next generation of nuclear and particle physics experiments at high beam luminosity. Silicon Carbide detectors for Intense Luminosity Investigations and Applications (SiCILIA) is a project starting as a collaboration between the Italian National Institute of Nuclear Physics (INFN) and IMM-CNR, aiming at the realization of innovative detection systems based on SiC. In this paper, we discuss the main features of silicon carbide as a material and its potential application in the field of particles and photons detectors, the project structure and the strategies used for the prototype realization, and the first results concerning prototype production and their performance.

## 1. Silicon Carbide

Silicon carbide (SiC) is a semiconductor with a wide, indirect bandgap. It is one of the hardest materials present in nature. The strong bonds determine a large bandgap, implying a high refractive index and a broad transparency over the visible spectrum. Today, these properties make SiC an ideal material in space applications and for the realization of ultra-lightweight mirrors [1] and bearings [2]. SiC also has extreme thermal stability, sublimating at 2830 °C. Its thermal conductivity is close to the value of copper and is three times higher than Si. Other properties, such as transparency to visible light, ultraviolet (UV) wavelength absorption, radiation hardness and biocompatibility, make this material attractive for alternative application fields, such as high-temperature electronics, biomedical sensors, UV photo-sensors, and charged particle and X-ray detectors.

Silicon carbide shows a large variation in crystal lattices according to the stacking sequence of the atoms in the crystalline lattice. This property is known as “polytypism”, and the stacking sequence causes the presence of both hexagonal and cubic lattice sites. Though SiC exists in more than 200 different polytypes [3], the 3C, 4H, and 6H structures are the most common and the most popular for microelectronics applications. Each polytype has its own physical properties, such as the energy bandgap, which ranged from 2.36 eV in 3C to 3.23 eV in 4H [4]. Among all the SiC polytypes, 4H–SiC is considered to be the most appropriate for high-power, high-frequency, and high-temperature applications in microelectronics, as it has the widest bandgap and an almost isotropic electronic mobility.

Table 1 lists the main physical properties of SiC and Si at room temperature. Among the tabulated properties, some make SiC a very singular material with respect to Si, such as its three times larger bandgap, the three times higher thermal conductivity, and ten times higher breakdown electric field strength. So, it is possible to affirm that SiC has a high potential to reach the market in a wide range of device applications. Unlike other semiconductor materials of technological interest, silicon carbide does not have a liquid phase. The only way to grow silicon carbide for device processing is by means of gaseous phases.

The high-quality material used for device application is typically grown epitaxially by a Chemical Vapor Deposition (CVD) process. Epitaxy allows a highly precise control of thickness, doping, and homogeneity of crystal films. The epitaxial growth of SiC films is typically realized in horizontal hot-wall reactors in a low-pressure regime. The growth of 4H–SiC polytype requires temperatures ranging from 1600 to 1650 °C.

During the CVD process, the dopants are also provided by means of gaseous precursors (such as Nitrogen N_2_ for n-type doping and Trimethylaluminium TMA Al_2_(CH_3_)_6_ for p-type doping). The incorporation of dopants is not only controlled by the concentration of the dopant gases, but also by means of a site competition effect (i.e., by the Si/C ratio of the source precursors). This is because there is a preferential occupation of the carbon sites for donors and of the silicon sites for acceptors, respectively [5].

An important aspect of any semiconductor crystal is the presence of lattice defects, which often limits the performance of the device, significantly hindering the operation and degrading its properties by introducing new electronic levels in the forbidden gap that interfere with charge carriers in the valence and conduction band.

A thorough characterization of the defects, together with an evaluation of their influence on the device performance and a strategy to evaluate their density are therefore key elements of a production process starting with the production of new crystals and ending with the production of new devices.

To obtain a high-quality material, one uses as seeds substrate with the lowest possible defect concentration and an epitaxial process able to reduce the density of defects present in the substrates. A stacking faults (SF), for example, one of the main defects of silicon carbide crystals, comes from a particular kind of defect of the substrate, called basal plane dislocation (BPD). This defect propagates into the epilayers from the substrate acting as source for SF formation. The density of SFs should be very low for electronic devices, because these defects are carrier lifetime *killers*. Many studies have been performed to improve the quality of epitaxial SiC films, such as the work of F. La Via et al. [6]. They increased the growth rate by increasing the silane flow combined with the introduction of hydrogen chloride in the deposition chamber. This process produced a higher deposition rate (more than a factor 10) with a low surface roughness (about 0.3 nm RMS), a high minority carrier lifetime (about 1 μs for a 100 µm epitaxy), and good thickness and doping uniformity [7,8].

The same authors [9,10] in a different approach, have grown 4H–SiC epitaxial layers using ethylene as carbon precursor together with trichlorosilane (TCS, SiHCl_3_) as a silicon precursor. TCS avoids the homogeneous nucleation of silicon droplets in the gas phase, shifting from Si to SiCl_2_ the dominant Si-containing species for the growth. While atomic Si is responsible for the homogeneous nucleation of silicon droplets in the gas phase, SiCl_2_ is very stable and suppresses homogeneous gas phase nucleation of Si during the growth.

Some growth parameters, such as the growth rate condition, have a strong influence on the defect formation or annihilation [11]. For example, a clear decrease in the basal plane dislocations density was observed with increasing the growth rate. This reduction of BPDs also produces a low density of single Shockley faults (SSFs), as reported by Kimoto [12]. SSFs are the main limit hindering the commercialization of efficient and stable bipolar devices. It has been observed that SFs induce a reduction of carrier lifetime and an increase of the leakage current [13].

SiC epitaxial layers are typically characterized by different types of surface defects, such as carrot defects [14,15,16], pit defects [17,18], triangular defects [19], down-fall, and other typologies [20]. The formation mechanism of these defects is not completely known, but it is clear that they are due to unoptimized growth conditions, because their density is correlated to substrate crystal quality and to the growth conditions. The presence of macroscopic defects has an influence on the leakage current and on the blocking voltage of a device [12], determining an increase of the former and a reduction of the latter in conditions of high defect density.

### Silicon Carbide as Particle Detector Material

The device structures typically used for the realization of a detector are semiconductor junctions (p^+^/n, n^+^/p, or a p^+^/i/n^+^) operating under reverse bias. A space charge region is formed, and the p^+^ or n^+^ regions or the metal layer act as electrodes. The lowest concentration of impurities and defects is required in order to avoid a reduction in the current signal amplitude due to the recombination of electron–hole pairs. The SiC wide bandgap reduces significantly the rate of thermally generated charge carriers, reducing the leakage current and the noise level. On the other hand, it could also represent a disadvantage, because a particle with a definite energy, ideally transforming all its energy for the production of electron–hole pairs, generates about twice the amount of charge carriers in Si than in SiC. Therefore, detectors based on SiC have lower pulse amplitudes. However, for small signals, the reduction of the noise level overcompensates for the reduction of the signal level so that the overall signal-to-noise ratio (SNR) is improved for SiC detectors. Furthermore, SiC detectors still have a high SNR at higher temperatures with respect to Si devices, unless external cooling is used to decrease the intrinsic carrier level [21].

The radiation damage can affect various properties of a detector, such as the leakage current and the charge collection efficiency (CCE), due to the removal of free carriers from the active region of the device. Radiation hardness is the robustness of these device parameters against high doses of particle irradiation [22]. Silicon carbide, due to its wide bandgap and chemical bonds strength, has been considered as an alternative to silicon for the production of radiation hard detectors [23,24,25,26].

A further important aspect, in connection with the large bandgap, is SiC’s insensitivity to photons in the visible range, making SiC devices very promising for neutrons and charged particles detection in a plasma environment [27].

## 2. The Silicon Carbide Detectors for Intense Luminosity Investigations and Applications (SiCILIA) Project Approach

In view of the potential application of SiC as a radiation hard material for detector implementation, and thanks to the request for several new and ambitious projects by the Italian National Institute of Nuclear Physics (INFN) in fundamental and applied nuclear physics (NUMEN [28,29], NuReLP [30], ELIMED [31], FAZIA [32], etc.), a cooperation has been started between INFN and IMM-CNR for a common research and development activity regarding silicon carbide technology. A specific project, named SiCILIA, has been totally funded by the INFN.

All the cited projects require radiation hard detectors with excellent performance in terms of stability, energy resolution, timing, and insensitivity to the visible light. Some of them also require a relatively large detection area—larger than 1 cm^2^, with thicknesses in the range between 50 and 1000 µm, without dead layers—in order to guarantee the implementation of more complex detection systems, such as telescope detectors, which are useful in several applications (NUMEN, FAZIA). In a detector telescope, two detector stages are placed along the particle trajectory. The particle releases part of its energy in the first, thinner stage (Δ*E*), and it is then stopped by the second, thicker stage, which measures its residual energy (*E*) [33].

Thicknesses, dimensions (detection area), and manufacturing technologies are the actual technological limits on SiC material processing. Most of the commercial SiC devices are realized through Schottky junctions implementation. This project wants to overcome these limits by using two main strategies: (i) the use of epitaxial layers grown on custom 4H–SiC substrates, widely available on the global SiC market, for the implementation of Δ*E* detectors (50–300 µm thick); and (ii) the use of semi-insulating SiC substrates (300–1000 µm thick) for the *E* detectors. So, the general idea of the project is the development of innovative processes for the production of relatively large area SiC detectors, both thin (Δ*E*) and thick (*E*), as required for specific nuclear physics experiments. Two device structures have been developed: Schottky diodes and p^+^-n^−^ junctions.

Today, Schottky junctions [34] represent the state of art for SiC device and detectors. We want to push forward the limits of this technology, especially in relation to the detector thickness and active area. The p^+^-n^−^ structure represents instead a novel and very promising solution for SiC detectors, in analogy to similar junctions based on silicon.

After a first pre-production of devices, the project schedule foresees a characterization with several kinds of ionizing radiations, light- and heavy-ions, electrons, neutrons, and photons. Standard and customized low-noise electronics will be designed and tested. Energy resolution, charge collection efficiency, timing, linearity, and pulse-shape discrimination capability will be also determined.

## 3. Status and First Results

The first step of the project was the growth of the materials that in the second step are used for the detector implementation.

### 3.1. Epitaxial Growths and Characterization of Materials

The strategy of the project was the use of epitaxially grown material as the active layer for the realization of Δ*E* detector and the use of semi-insulating thick 4H–SiC material for the *E* detector. The quality of 4H–SiC epitaxial material is nowadays very high, considering the achievements of the last decades in the growth of material.

The epitaxial growth was performed in a horizontal hot-wall reactor (LPE-PE106) [35]; trichlorosilane (SiHCl_3_ or TCS), ethylene (C_2_H_4_), and hydrogen (H_2_) were used as silicon and carbon precursors and gas carrier, respectively. Nitrogen (N_2_) was used for n-type doping and Trimethylaluminium TMA Al_2_(CH_3_)_6_ for p-type doping. The processes were realized in a low-pressure regime at a temperature of 1630 °C. Two different growth rate values were used for the deposition (90 μm/h and 60 μm/h). The growth of a thick low-doped n-type layer (100 µm, N_D_ = 8 × 10^13^ cm^−3^) and a thin p-type highly doped layer (0.3 µm, N_A_ = 1 × 10^19^ cm^−3^) was performed for the p–n junction detector.

The materials were then accurately characterized with different techniques: the inspection with Candela CS920 by KLA-Tencor [36] scan was used to evaluate the presence of the surface defects (droplet, carrots, triangles, micropit, etc.) characterizing the epitaxial layers. Room-temperature micro-photoluminescence (RT-μPL) and time-resolved photoluminescence (TRPL) were performed to analyze the crystal quality of the materials.

The tool Candela CS920 by KLA-Tencor is an inspection system that allows for surface defects detection and photoluminescence (PL) inspection in a single platform. Through the dual laser implementation (UV and optical lasers) and several detectors at different angles, different signal channels are acquired at the same time. The PL signal, the scattered and reflected light detected with a single acquisition step, is able to collect all the defect features on the analyzed sample. The detection of macro- and microdefects and the automatic classification is obtained through a cross-correlation between the channels with different properties (wavelength of the laser, angle with the surface, amplitude of the scattered, and specular light). The acquired signals are filtered by customized rule-based binning that allows us to create a reliable defect classification. The high spatial resolution obtained in the whole wafer characterization allows for detailed comparison between the signals coming from the different channels. The detection of features belonging to the same defect, observed in different channels, could be the key for a deep comprehension of the SiC crystal quality, and consequently, it provides a valuable suggestion for improving the quality of the epitaxial layer by reducing the defect density.

**Figure 1:**
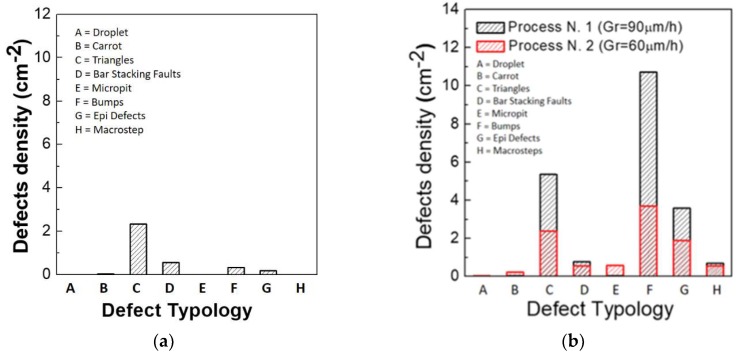
Surface defects acquired with a Candela scan on 10 µm 4H–SiC epitaxy (**a**). Evaluation of defects density by Candela scan (**b**) on 100 µm 4H–SiC epitaxies grown with two process typologies (at 90 μm/h and 60 μm/h).

In Figure 1a, the defect density obtained by Candela analysis for the 10 µm thick epitaxy is reported. It is possible to observe from these data that the main defects are the triangles, followed by bar stacking faults. The total density of defects is around 3/cm^2^, and these defects have been observed essentially on the edge of the wafers. With this defect density, a maximum yield of 65–70% can be achieved for the 1 cm^2^ detectors. In Figure 1b, the defect density, evaluated by Candela analysis, is reported for two representative 4H–SiC epitaxial films (100 μm) grown with two different growth rate conditions (90 μm/h and 60 μm/h). The defects density is lower in the sample grown at 60 μm/h, indicating that a fast deposition induces the formation of surface defects, such as triangles, bumps, and epi defects. In both cases, the defect density is much higher than in the 10 µm thick epitaxy. This effect is partly due to the lower quality of the 6-inch substrates used for the 100 µm thick epitaxy with respect to the 4-inch substrates used for the 10 µm thick epitaxy and partly due to the large thickness of the epitaxial layer that produces a higher defect density, as previously observed on 3-inch substrates [37].

The room-temperature micro-photoluminescence (RT-µPL) analyses were performed with a HR800 integrated system by Horiba Jobin Yvon in a back-scattering configuration with a microscope confocally coupled to a 800 mm focal length spectrograph. The excitation wavelength was supplied by a He–Cd laser (with 325 nm wavelength and 5 mW power) and was focused on the sample by a 40× objective with a numerical aperture of 0.45. The PL signal was dispersed by a 300 grooves/mm kinematic grating. A silicon CCD with size of 1024 × 256 pixels was used as the detector.

**Figure 2:**
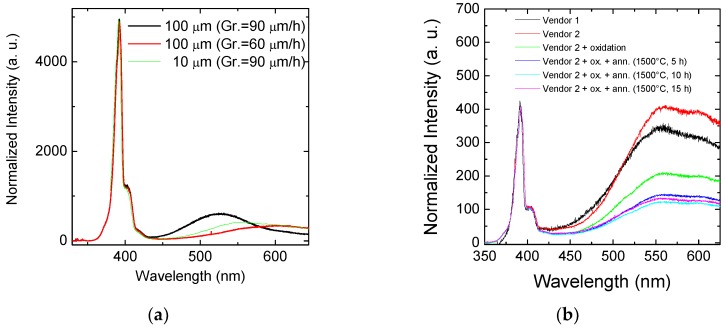
(**a**) Room-temperature micro-photoluminescence analysis on 100 µm 4H–SiC epitaxies grown with two process typologies (at 90 μm/h and 60 μm/h) and 10 µ; (**b**) room-temperature photoluminescence (RTPL) analysis on semi-insulating substrates for *E* detector, after oxidation and thermal treatments at 1500 °C for different annealing times.

In Figure 2a, the room-temperature photoluminescence analyses on the 10 µm and 100 µm epitaxies grown at 90 μm/h and 60 μm/h are shown. In the photoluminescence spectra, the emission between 350–420 nm is due to the bandgap emission, while the band emission between 450 and 600 nm is related to the point defects. The photoluminescence analyses indicate a better quality for the epitaxy grown at 60 μm/h, with a lower point defect density. Almost the same point defect peak is observed in the 10 µm epitaxy. The density of point defects is extremely important in this application because a high density of these defects can increase the leakage current of the junction, increasing the noise of the detector, and can also greatly reduce the carrier lifetime of the devices, with a strong reduction of the signal intensity. Therefore, these two effects can strongly affect the SNR ratio of the detectors.

Furthermore, the point defect peak in Figure 2a has a different position for the three different processes. This means that the relative ratio of the different point defects (Z1/Z2, EH6/7, …) can vary in the three processes, thus affecting the carrier lifetime [38].

Figure 2b shows a room-temperature photoluminescence analysis performed on semi-insulating materials that will be used for the realization of *E* detectors. The quality of the as-grown material is lower with respect to the epitaxial material, as is demonstrated by the higher intensity of the point defect band that can be observed in the PL spectrum between 450 nm and 600 nm. So, in order to improve the quality of such materials and increase the carrier lifetime, we performed an oxidation treatment and subsequent annealing processes (at 1500 °C for different durations: 5 h, 10 h, and 15 h) according to some published works [39]. This kind of process can introduce a large amount of carbon interstitial by oxidation, and the high-temperature annealing diffuses these interstitials and annihilates the carbon vacancies that are present in the substrate.

From the data reported in Figure 2b, it is possible to observe that the oxidation and the following high-temperature diffusion is effective in the reduction of these point defects, but even for the longest annealing of 15 h at 1500 °C, the point defect peak is still remarkable. Longer annealing times are not feasible for the production of these detectors, and thus, this high density of point defects can represent a problem for the production of detectors from intrinsic wafers.

Samples achieved by several vendors have been tested but, as also reported in Figure 2b, the starting density of the point defects in these wafers is always much higher than in the epitaxial materials.

The time-resolved PL analyses were performed using a HR800 integrated system by Horiba Jobin Yvon with a 10× objective. The excitation wavelength was supplied by a pulsed NanoLED featuring a 375 nm wavelength and 5 ns pulse width. With this wavelength, all the substrate can be penetrated and analyzed. The detector was a photomultiplier working in a photon-counting regime. In order to perform the carrier lifetime measurements, the decay time of the 392 nm (with a spectral width of 10 nm) band was used. Spectra were acquired using a time-correlated single photon-counting system by Horiba Jobin Yvon and analyzed by DAQ software.

The thermal oxidation of SiC eliminates specific carrier traps, such as Z1/Z2 and EH6/7, which are related in particular to carbon vacancy, which determines an increase of carrier lifetime. This is beneficial for reducing the on-resistance in bipolar devices and for the detector operations. The PL spectra in Figure 2b also show the improvement of the material quality after the oxidation and annealing processes with the reduction of intensity of the defects band. On the semi-insulating materials (as-grown and treated), we also performed the time-resolved PL analysis to evaluate the lifetime of carriers.

**Figure 3:**
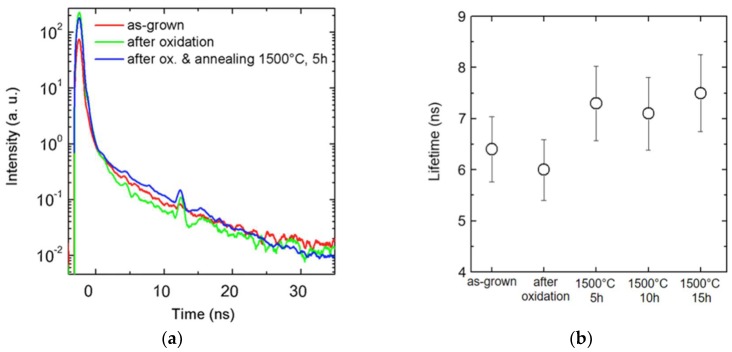
(**a**) Time-resolved photoluminescence (TRPL) analysis on semi-insulating substrates, for *E* detector, after oxidation and thermal treatments at 1500 °C for different exposure periods. TRPL spectra (**a**) and lifetime values obtained (**b**).

Figure 3a,b report the time-resolved spectra and the evaluated lifetimes, respectively. A lifetime of about 6 ns was evaluated for the as-grown semi-insulating material and for the same material after oxidation; a little increase, at about 7.5 ns, was observed after oxidation and thermal annealing, thus demonstrating the effect of the annealing process. However, longer annealing times are probably needed in order to improve the quality of the material along the whole thickness of the substrate. For this reason, several new intrinsic materials coming from different producers will be tested to find the best solution in terms of lower point defect density and longer carrier lifetime.

### 3.2. Prototypes Processing

After studying the material processing, as discussed in the previous paragraphs, a number of prototype devices (p–n junctions) have been manufactured in the STM clean rooms from 10 µm epi-layers, in order to investigate the processes for the production of ∆*E* detectors.

**Figure 4:**
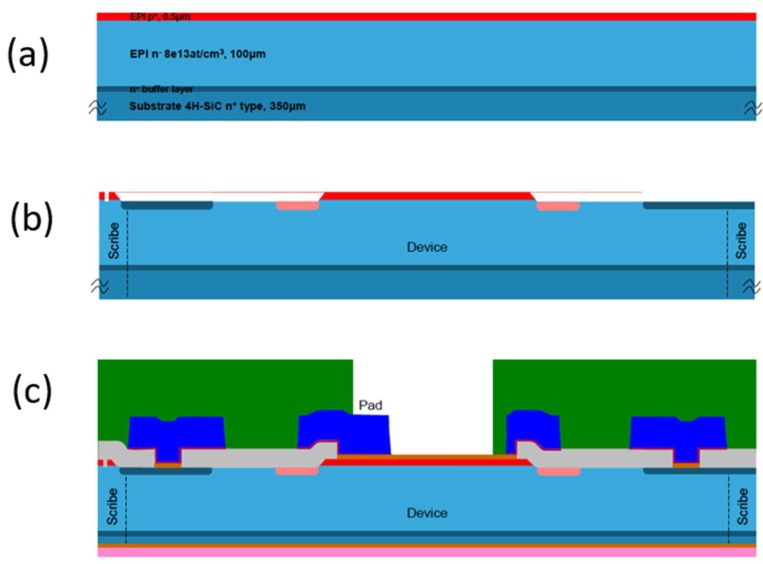
(**a**) Epitaxial structure of a ∆*E* detector; (**b**) etching of the p^+^ region and definition of the edge structures; (**c**) insulation oxide, front metallization, substrate thickness reduction, and backside metallization.

The first step of the processing is the epitaxy of the double layer necessary for the p^+^/n junction. A p^+^ layer 0.3–0.5 µm thick with a doping concentration of the order of 10^18^–10^19^/cm^3^ was grown over the n^−^ epi-layer with a doping concentration in the range of 5–8 × 10^13^/cm^3^ (Figure 4a). After this step, a first photolithography for the definition of the detector area by a plasma etching was performed. Then, a second lithography was performed for the construction of the edge structures aimed at reducing the electric field at the device borders. In particular, these edge structures were performed by ion implantation at a temperature of 400–500 °C followed by high-temperature annealing (1600–1800 °C) to recover the crystalline structure of the SiC implanted regions (Figure 4b) or using a metal field plate or finally using several floating rings realized with the p^+^ epitaxial layer. All these structures were realized on the same wafers to compare the breakdown of efficiency of the different structures. Even different implantation doses have been tested to see the dependence on the dose of the breakdown voltage.

During the annealing steps, a process step aimed at increasing the minority carrier lifetime of the epitaxial layer was also performed at a temperature of 1500 °C. The process continued with the deposition of an isolation oxide and the opening of the contacts with a further photolithographic process. Then, the front metallization (Ni) was deposited, defined with a further photolithographic process and subsequently annealed to form a good ohmic contact on the p^+^ region. Only on the periphery of the detector, a thicker layer of Ti and Al was deposited for the bonding.

Finally, the substrate was reduced by a mechanical process from the backside, and the ohmic contact was formed by a titanium/nickel/gold deposition (Figure 4d) [40].

In the case of the *E* detector, an intrinsic wafer was used instead of the usual n^+^ substrate. Thanks to this choice, the detector featured a relatively thick un-doped layer (0.5–1 mm). For this type of detector, a thin p^+^ epitaxial layer on the front and a thin n^+^ epitaxial layer on the backside should be grown for the realization of the junction and of the back contact. After these epitaxial layers grew, the process was essentially the same as for the previous detector, except for the thickness reduction of the substrate.

## 4. 10 μm Thick Detectors

In order to test and optimize the fabrication processes, a first production run on 4-inch wafers (with a 10 µm thick epi-layer) was processed by STM.

**Figure 5:**
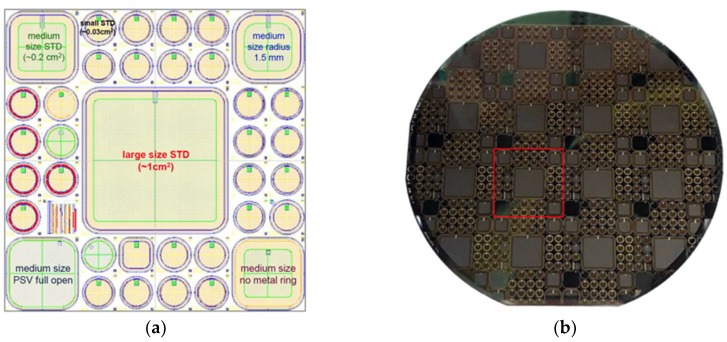
(**a**) Single step layout view; (**b**) typical 4-inch wafer, single step highlighted.

In Figure 5a, the layout of the different detectors is shown. In this first run, different edge structures were tested using junction termination extension (JTE) implanted rings (with three different doses), a metal field plate, and 16 floating rings realized using the p^+^ epitaxial layer. Figure 5b shows an image of a typical 4-inch wafer containing several devices.

The goal of this first experiment was to test the different edge structures and the fabrication process. The testing process can be used subsequently also for the 100 and the thicker detectors.

**Figure 6:**
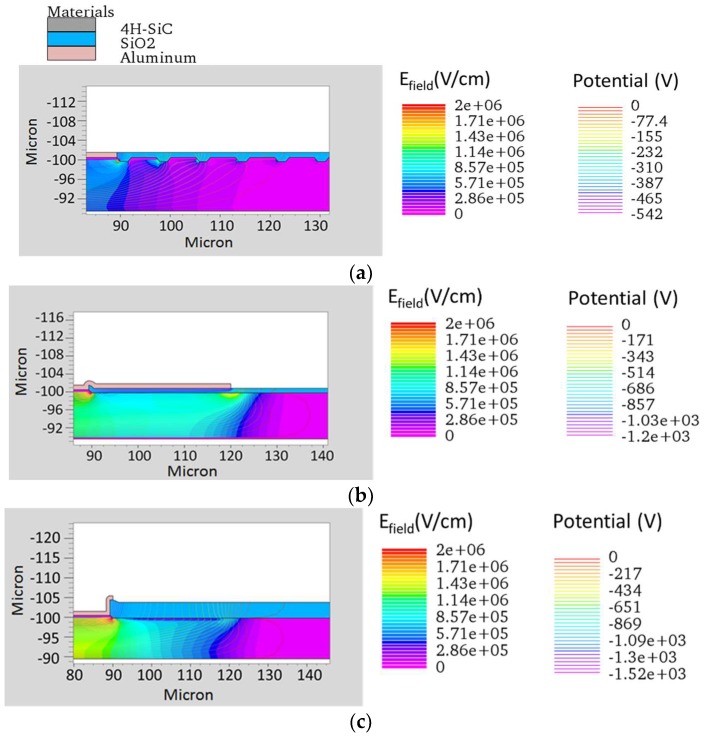
(**a**) Simulations of the edge structure with 16 epitaxial rings; (**b**) with field plate; (**c**) with p^−^ implanted ring. All the edge terminations have been optimized for 100 μm epi thickness but are shown for the 10 μm epi layer, close to the breakdown voltage.

All these edge structures were simulated by Silvaco Athena code. Several layout solutions and implanted doses were tested experimentally in order to estimate the breakdown voltage and evaluate the influence of the different edge structures on the detector performance and reliability in working conditions under high-energy ion fluxes. All the optimization work of the edge structure was done using the 100 µm thick epitaxy. Using these optimized structures, the simulations for the 10 µm thick detectors were also performed in order to compare the results with the experimental values (Figure 6).

**Figure 7:**
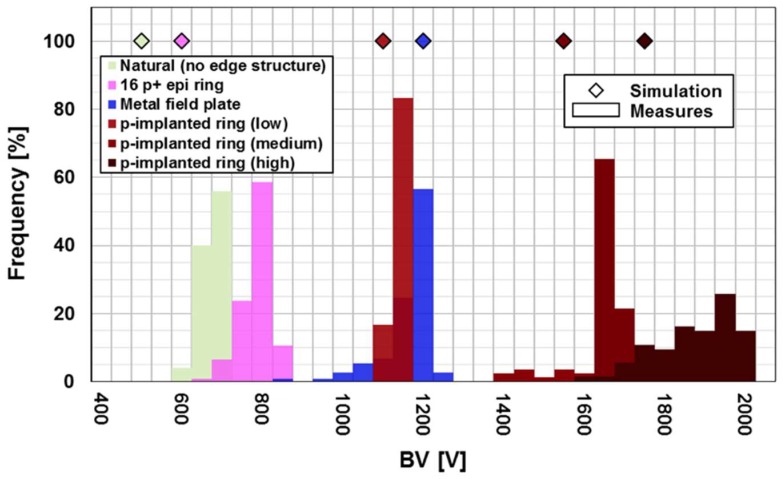
Comparison between the experimental distributions of the breakdown voltages of the detectors with 10 µm epitaxy and the simulations. A good agreement is observed for all the edge structures with the exception of the detectors without edge structure and the detectors with 16 epi rings.

From the first results shown in Figure 7, it is possible to observe that the simulated breakdown voltages (BV) of the different edge structures are in good agreement with the experimental results. For the case without edge structure and the one with 16 p^+^ epi rings, the disagreement is probably due to the particular etching profile of the p^+^ epi layer that was not perfectly simulated. Moreover, the distribution of the breakdown voltages for the different structures seems extremely narrow with the only exception being the p^−^ implanted ring with the highest dose.

From these data it appears that the p^−^ implanted ring with the highest dose shows the highest breakdown voltage, but in any case, all the edge termination structures can be used for the complete depletion of the epitaxial layer.

**Figure 8:**
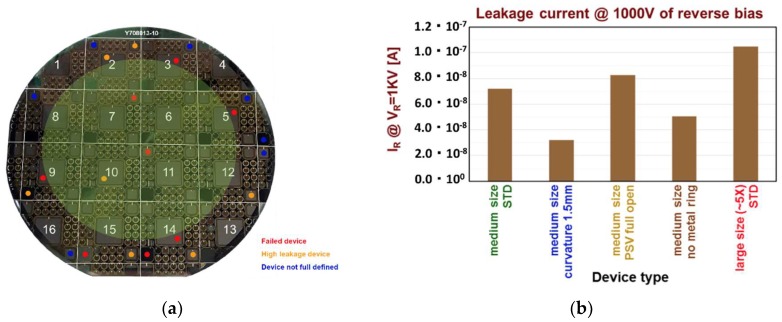
(**a**) Image of a typical wafer with the indications of the failed and of the high-leakage devices. The majority of the failed devices are on the edge of the wafer where the majority of defects are present; (**b**) the leakage currents of the good devices do not show any dependence on the particular edge structure.

From the point of view of the production yield (Figure 8a), no effect of the edge structure can be observed. Obviously, the yield decreases for the larger diodes, and we observe also an increase of the failure towards the edge of the wafer in accordance with the increase of the crystallographic defects, as also observed in the Candela maps. The average leakage currents (Figure 8b), with the exclusion of the failed devices, are also insensitive to the different edge structures.

**Figure 9:**
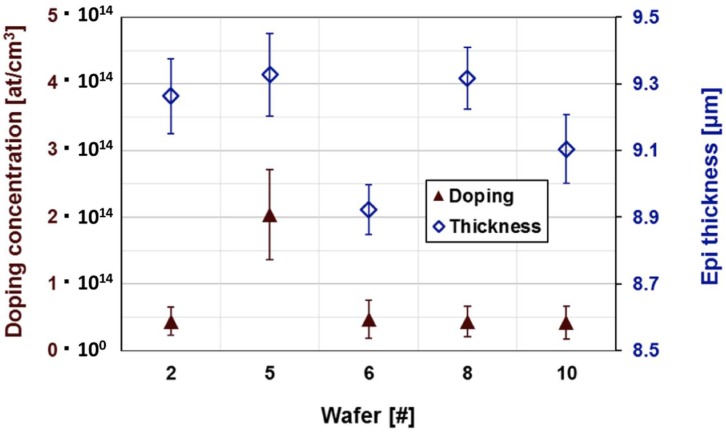
Doping concentration and epitaxial layer thickness as obtained by C–V measurements on several detectors on different wafers.

Finally, the thickness and the doping of the epitaxial layers were measured on the different wafers by C–V measurements (Figure 9). The results show a good uniformity and reproducibility of the doping at these low concentrations and a good uniformity and reproducibility of the thickness.

## 5. Detector Testing: First Results

**Figure 10:**
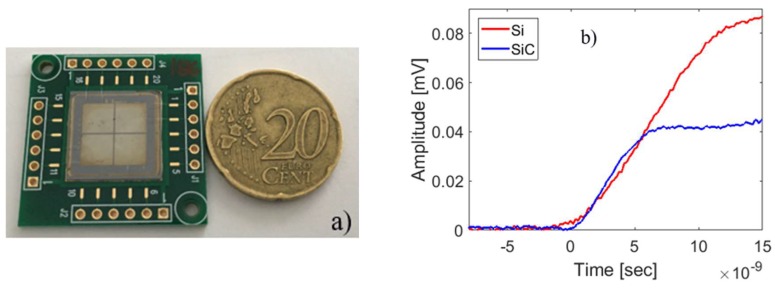
(**a**) Image of large area 100 µm SiC detectors; (**b**) signals at the output of preamplifier for silicon and SiC detectors.

**Figure 11:**
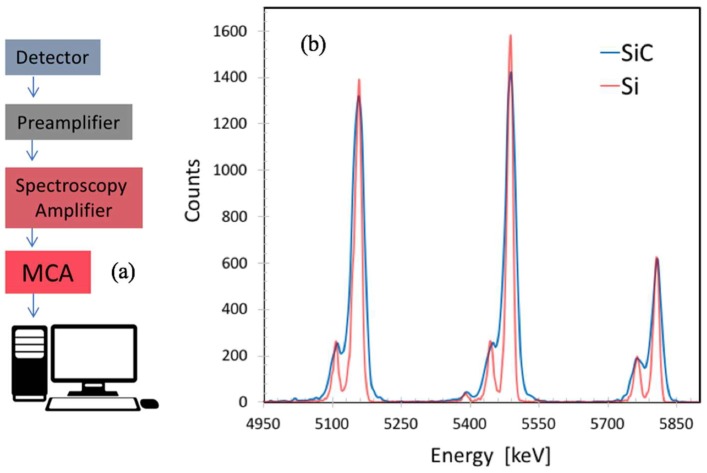
(**a**) Spectroscopic electronic chain used for the detectors test; (**b**) energy spectrum of alpha source (^239^Pu, ^241^Am, ^244^Cm) measured by Hamamatsu S3590 Si detector and by our SiC prototypes. The average energy spread obtained for the Si detector is of the order of 0.22%, while we obtained about 0.4% for SiC.

Using the same process illustrated for the 10 µm thick detectors, 100 µm thick detectors were also been produced (see Figure 10); they have an active area of 1 × 1 cm^2^ with a cathode segmented into four parts. In this last case, only the edge structure with the 16 floating rings around each detector was adopted. These devices were tested in order to study their performance using a radioactive alpha source and a standard spectroscopic electronic chain (Figure 11a). It consists of a charge preamplifier from the ASCOM company [41] (45 mV/MeV of gain, 600 µs of decay time, 1.5 keV at zero pF, and 12 eV/pF of slope as noise figure) and of a spectroscopy amplifier by ORTEC corporation (mod. 572, settled with 2 µs of shaping time). The signal after formation has been acquired by an ORTEC multi-channel analyzer to perform the energy spectra.

For a direct comparison with a state-of-the-art detector, the whole chain was also used with an Hamamatzu S3590-06 Silicon detector, 300 µm thick, 1 × 1 cm^2^ of active area. Both detectors were polarized with a reverse bias (600 V for SiC and 150 V for Si) in order to guarantee the best performance. In Figure 11b, typical preamplifier signals produced by alpha particles from an ^241^Am source are plotted for both detector types. Figure 11b shows the alpha particle energy spectrum for a mixed nuclides source (^239^Pu, ^241^Am, ^244^Cm). It can be noticed that the signals from the SiC detectors are about two times smaller than that for silicon, due to the difference in the energy bandgap; SiC also exhibits an energy resolution for alphas coming from ^241^Am of 42.8 ± 1.1 keV (FWHM), while the silicon detector gives under the same conditions about 21.4 ± 0.8 keV.

Such differences can be partially due to differences in the dead layers of the entrance region of the detectors, in the mean energy per e–h pair creation, to the fluctuations on charge collection, and also to the electronic noise contribution.

The Hamamatsu detector is specially designed for soft X-ray applications, and then, the dead layers have been specially minimized (0.32 µm Si-equivalent [42]). Our prototypes, on the other hand, present a first layer of metal (composed by 22 nm of titanium, 40 nm of aluminum, and 60 nm of nickel), followed by an inert p^+^ layer (0.3 µm thick). Such regions introduce an overall energy straggling of about 10.6 keV (FWHM) for the silicon and 17 keV (FWHM) for the SiC detector, respectively (SRIM [43] calculation).

The electronic noise contribution was evaluated performing an ad-hoc measurement: the electronic chain was stimulated by using a very precise pulse generator connected to the test input of the preamplifier. In such a condition, it is possible to determine the energy spread due to electronics, which has been evaluated in 5.7 channels (*FWHM^Ele^*). Such value gives a contribution of 37.9 keV for SiC and 17.1 keV for silicon.

In Table 2, we list the overall energy resolutions for both detectors after subtracting the contributions of the dead layers and the electronic noise. Such values (which includes the statistical fluctuations, the charge collection effects, and the noise due to the leakage current) are very close to their statistical limits due to the fluctuations of the number of pairs produced by the impinging radiation, assuming a Fano factor F = 0.11 [33] (usual for semiconductors) for both silicon and SiC detectors.

## 6. Summary

In this paper, the SiCILIA approach for a new class of silicon carbide detector is presented, and the process and the first results are described in detail. In relation to the *E* detector made by using thick epitaxial layers grown on custom 4H–SiC substrates, the defects concentration can be kept under control, lowering the growth rate (60 μm/h); fast deposition induces the formation of surface epi defects and point defects. With this process, a good yield of large detectors has been obtained.

Concerning the *E* detector, it has been observed that the intrinsic wafers have a high concentration of point defects. These defects induce a low carrier lifetime. An oxidation process with a subsequent diffusion process of carbon interstitial has been tested to reduce these defects, but the effect on the carrier lifetime is marginal. New processes should be tested to solve this problem that induces a low charge collection in the *E* detector.

The 10 µm detectors have been used to test the process and to test the edge structures. The used fabrication process produces a good yield and good characteristics of the detectors. Also, a perfect agreement between the simulations and the breakdown experimental measurements has been observed.

Prototypes were tested, in comparison with a commercial silicon device, in order to evaluate their performance. These exhibit excellent performances in terms of timing and energy resolution.

These preliminary results show that 4H–SiC has a great potentiality in the field of heavy-ion detectors for high-resolution, large SNR, and strong radiation hardness.

## Figures and Tables

**Table 1:** Comparison between silicon versus SiC properties.

Properties	4H–SiC	Si
E_gap_ [eV]	3.23	1.12
E_breakdown_ [V/cm]	3–4 × 10^6^	3 × 10^5^
µ_e_ [cm^2^/Vs]	800	1450
µ_h_ [cm^2^/Vs]	115	450
V_saturation_ [cm/s]	2 × 10^7^	0.8 × 10^6^
Z	14/6	14
ε_r_	9.7	11.9
E–h energy [eV]	7.6–8.4	3.6
Density [g/cm^3^]	3.22	2.33
Displacement *E* [eV]	30–40	13–15
Thermal Conductivity [W/cm·K]	4.9	1.5

**Table 2:** Silicon and SiC resolutions in comparison with their statistical limits.

Detectors	$FWHM^{det}\;\left[keV\right]$	$FWHM^{stat}\;\left[keV\right]$
SiC	10.3	5.0
Si	7.3	3.5
